# Clinicopathological and immunohistochemical analysis of spindle cell squamous cell carcinoma of the tongue: a rare case

**DOI:** 10.31744/einstein_journal/2019RC4610

**Published:** 2019-02-14

**Authors:** Diego Filipe Bezerra Silva, Hellen Bandeira de Pontes Santos, Jorge Esquiche León, Daliana Queiroga de Castro Gomes, Pollianna Muniz Alves, Cassiano Francisco Weege Nonaka

**Affiliations:** 1 Universidade Estadual da Paraíba, Campina Grande, PB, Brazil.; 2 Universidade Federal do Rio Grande do Norte, Natal, RN, Brazil.; 3 Faculdade de Odontologia de Ribeirão Preto, Universidade de São Paulo, Ribeirão Preto, SP, Brazil.

**Keywords:** Carcinoma, squamous cell, Mouth/pathology, Tongue neoplasms/diagnosis, Immunohistochemistry, Carcinoma de células escamosas, Boca/patologia, Neoplasias da língua/diagnóstico, Imuno-histoquímica

## Abstract

Spindle cell squamous cell carcinoma of the tongue is a rare variant of squamous cell carcinoma. This paper reports the case of a spindle cell squamous cell carcinoma of the tongue, in a 64-year-old male patient, and presents a review of the etiopathogenesis, clinicopathological and immunohistochemical features and treatment of the malignancy. The patient presented for evaluation of a painful swelling on his tongue. Extraoral examination revealed palpable submandibular and superior cervical lymph nodes. Based on the presumptive diagnoses of squamous cell carcinoma or malignant salivary gland neoplasm, an incisional biopsy was performed. Histopathological analysis showed a proliferation of atypical spindle cells, exhibiting extensive pleomorphism. Tumor cells were positive for vimentin, P53 and alpha-smooth muscle actin, focally positive for epithelial membrane antigen and P63, and negative for pan-cytokeratin (AE1/AE3), CK7, CD138, CD34, CD56, and S-100. The positivity index for Ki-67 was approximately 40%. The diagnosis of spindle cell squamous cell carcinoma was established and the patient was referred to a head and neck surgery service. In the oral cavity, spindle cell squamous cell carcinoma of the tongue is an aggressive variant of squamous cell carcinoma, which usually presents as an exophytic mass located on the tongue of elderly males. Due to its distinct histopathological characteristics, immunohistochemistry is a valuable and helpful tool to establish the diagnosis of spindle cell squamous cell carcinoma of the tongue.

## RESUMO

O carcinoma de células escamosas de células fusiformes da língua, é uma variante rara do carcinoma de células escamosas. Este trabalho relata o caso de um paciente do sexo masculino de 64 anos de idade com carcinoma de células escamosas de células fusiformes da língua. Foi feita uma revisão dos aspectos relacionados a etiopatogenia, características clinicopatológicas e imuno-histoquímicas, além do tratamento dessa neoplasia. O paciente procurou atendimento para avaliação de tumefação dolorosa na língua. O exame extraoral revelou linfonodos cervicais submandibulares e cervicais superiores palpáveis. Foi realizada biópsia incisional com base nas hipóteses diagnósticas de carcinoma de células escamosas ou neoplasia maligna de glândula salivar. A análise histopatológica demonstrou proliferação de células fusiformes atípicas, exibindo extenso pleomorfismo. As células tumorais foram positivas para vimentina, p53 e alfa-actina de músculo liso, focalmente positivas para antígeno de membrana epitelial e P63, e negativas para pancitoqueratina (AE1/AE3), CK7, CD138, CD34, CD56 e S-100. O índice de positividade para o Ki-67 foi de aproximadamente 40%. Foi feito o diagnóstico de carcinoma de células escamosas, de células fusiformes, e o paciente foi encaminhado para um serviço de cirurgia de cabeça e pescoço. Na cavidade oral, o carcinoma de células escamosas de células fusiformes da língua, é uma variante agressiva do carcinoma de células escamosas, que geralmente se apresenta como massa exofítica localizada na língua de homens idosos. Devido às suas distintas características histopatológicas, a imuno-histoquímica é uma ferramenta valiosa e útil para estabelecer o diagnóstico de carcinoma de células escamosas de células fusiformes da língua.

## INTRODUCTION

Spindle cell squamous cell carcinoma (SpCSCC) is a rare and aggressive variant of squamous cell carcinoma (SCC).^(^ ^1^ ^,^ ^2^ ^)^ The lesion reveals a biphasic aspect, characterized by proliferation of spindle cells with sarcomatous appearance associated with conventional SCC.^(^ ^1^ ^,^ ^2^ ^)^

Due to its heterogeneous nature, SpCSCC may be a real diagnostic challenge, even when representative samples are obtained from incisional biopsies. Thus, the microscopic differential diagnosis of SpCSCC ranges from malignant melanocytic lesions to sarcomas, low-grade tumors and reactive proliferative processes. Thus, immunohistochemistry is an important tool to establish the diagnosis of SpCSCC.^(^ ^3^ ^)^

The present study reported a case of oral SpCSCC and discussed aspects related to etiopathogenesis, clinicopathological and immunohistochemical features, differential diagnosis and treatment of the malignancy.

## CASE REPORT

**Figure 1:**
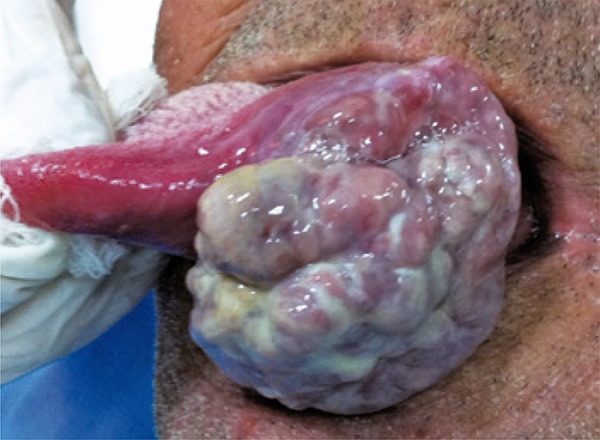
Large polypoid tumor located on the ventral tongue, on the left, with ulcerated areas, covered by fibrinopurulent pseudo membrane

A 64-year-old male smoker presented for evaluation of a painful swelling on his tongue, which had been identified 2 months earlier. On extraoral examination, palpable submandibular and superior cervical lymph nodes of the left side were observed. Intraoral examination revealed an extensive, exophytic and pedunculated mass on the ventral tongue, on the left, measuring approximately 6cm in diameter (Figure 1).

**Figure 2:**
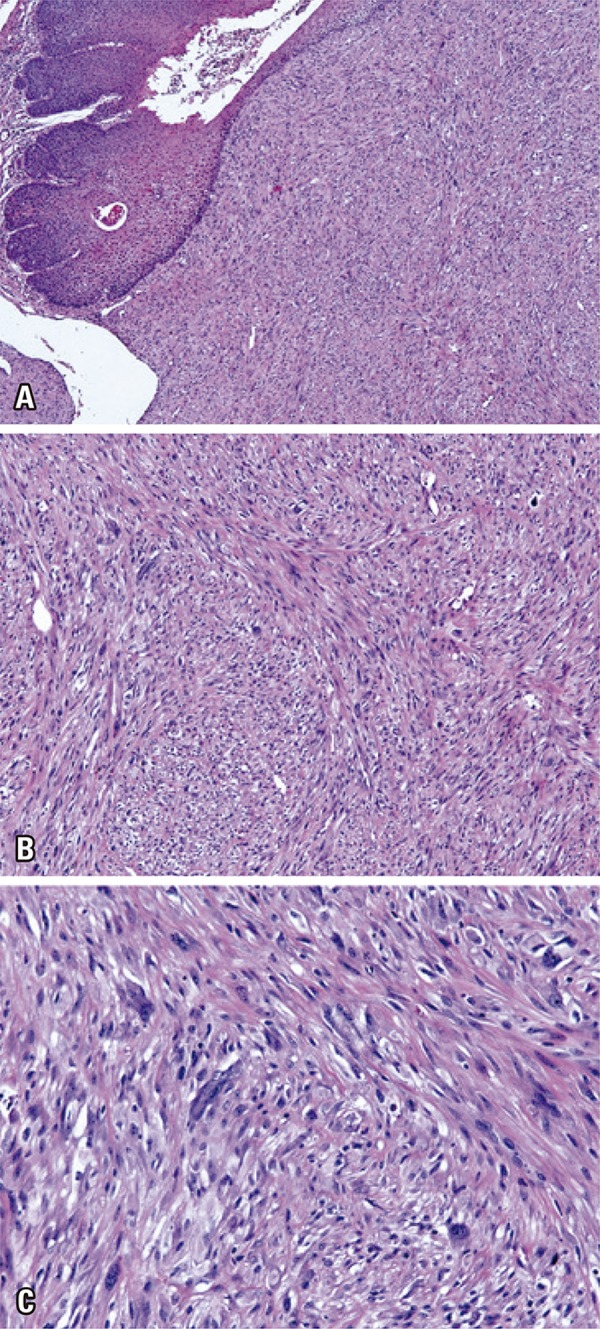
Histopathological aspects of the tumor. (A) Photomicrography shows parakeratinized stratified squamous epithelium, with areas of dysplasia and spindle cell proliferation in the underlying connective tissue (hematoxylin and eosin, X50). (B) Arrangement of spindle cells in fascicles in a fibrous stroma (hematoxylin and eosin, X100). (C) Cell atypia and mitotic figures in the spindle cell component (hematoxylin and eosin, X400)

An incisional biopsy was performed considering the presumptive diagnoses of SCC or malignant salivary gland neoplasm. Histopathological analysis showed a proliferation of atypical spindle, polygonal and epithelioid-like cells, arranged in fascicles. Superficially, the specimen revealed a stratified squamous epithelium with extensive discontinuous areas, varying degrees of dysplasia and foci of carcinoma *in situ,* but with no evident transition to the spindle-cell component (Figure 2).

**Figure 3:**
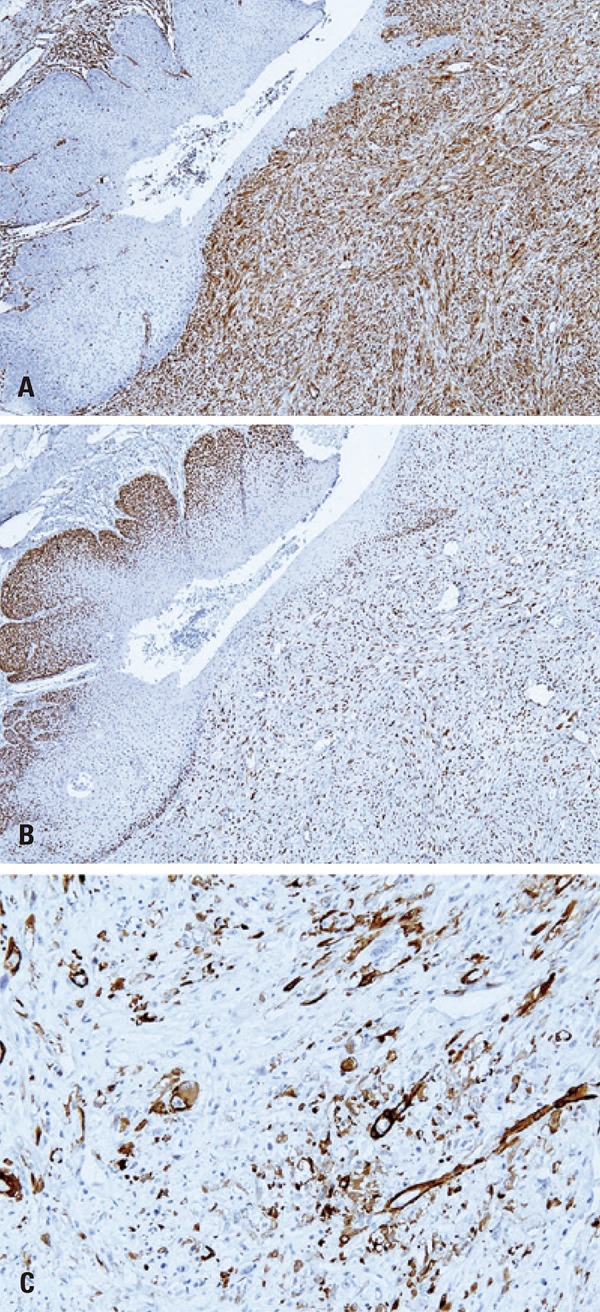
Immunohistochemical findings of the spindle cell squamous cell carcinoma. (A) Strong positivity of spindle cells to vimentin (Advance, X50). (B) Strong immunoreactivity for P53 in spindle cells and dysplastic epithelial cells (Advance, X50). (C) Spindle cells positive for alpha-smooth muscle actin (Advance, X200)

**Figure 4:**
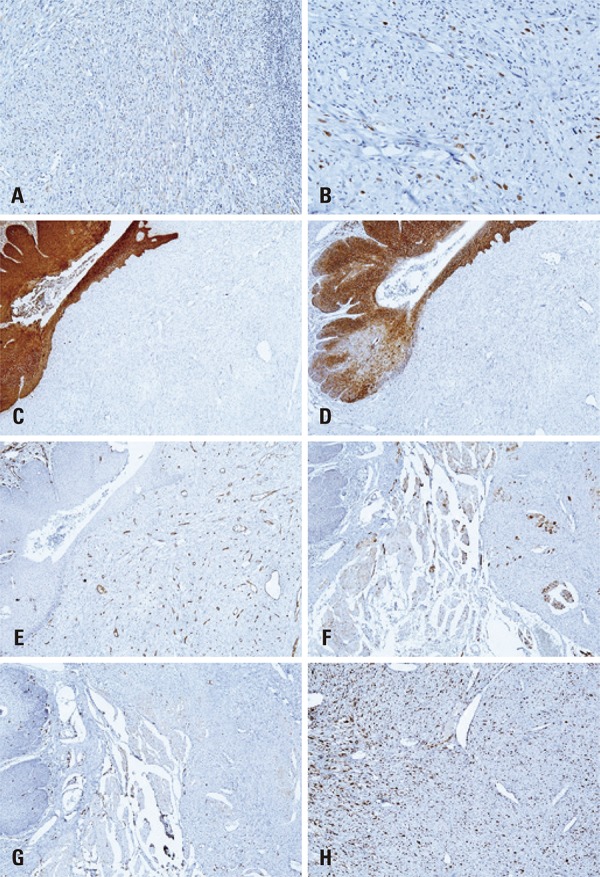
Additional immunohistochemical findings of the spindle cell squamous cell carcinoma. (A) Focal positivity of spindle cells to epithelial membrane antigen (Advance, X100). (B) Detail of positivity of neoplastic cells for P63 (Advance, X200). (C) Spindle cells negative for pan-cytokeratin (AE1/AE3) (Advance, X50). (D) Positivity of dysplastic epithelial cells to CD138 (Advance, X50). (E) Endothelial cells positive for CD34 (Advance, X50). (F) Negativity of neoplastic cells to CD56 (Advance, X100). (G) Negativity of neoplastic cells to S-100 protein (Advance, X50). (H) Ki-67 index of approximately 40% (Advance, X200)

Immunohistochemistry revealed tumor cells with strong positivity to vimentin and P53 (Figures 3A and 3B), positivity to alpha-smooth muscle actin (α-SMA) (Figure 3C) and focal positivity for epithelial membrane antigen (EMA) and P63. Otherwise, tumor cells were negative for pan-cytokeratin (AE1/AE3), CK7, CD138, CD34, CD56 and S-100 protein. Analysis of Ki-67 expression revealed a positivity index of approximately 40% (Figure 4). The diagnosis of SpCSCC was established and the patient was referred to a head and neck surgery service, but rejected the proposed treatment, abandoning it in its initial phase.

## DISCUSSION

Spindle cell squamous cell carcinoma is a rare malignancy, representing approximately 0.7% to 1.4% of oral SCC.^(^ ^2^ ^)^ This neoplasm is recognized as an example of a true epithelial-mesenchymal transition in human cancers.^(^ ^4^ ^)^ In the process of transformation of squamous cells into spindle cells, all criteria for epithelial-mesenchymal transition are fulfilled in SpCSCC. ^(^ ^1^ ^,^ ^4^ ^)^

One of the characteristics of epithelial-mesenchymal transition in SpCSCC is the decrease in E-cadherin expression, the main epithelial intercellular adhesion molecule, and increase in N-cadherin expression, responsible for the mobile phenotype of the cells.^(^ ^1^ ^,^ ^5^ ^)^ Other mechanisms include the morphological transition from epithelioid to a spindle cell phenotype; loss of cytokeratin and neo-expression of vimentin or co-expression of both, as observed in the current case; up-regulation of transcription repressors Snail, Slug, and Twist; and down-regulation of micro-RNAs, including miR-200 family and miR-205 in SpCSCC, in comparison to the normal epithelium and to conventional SCC.^(^ ^4^ ^,^ ^5^ ^)^

Spindle cell squamous cell carcinoma may occur in any part of the body, such as skin and breast.^(^ ^6^ ^)^ In the oral cavity, the tongue is the most common location, corresponding to nearly 42.2% of cases.^(^ ^6^ ^)^ A higher frequency of this neoplasm is reported in males and in the sixth decade of life.^(^ ^7^ ^)^ Smoking, alcoholism, previous radiotherapy in the region, and advanced age are factors that have been related to SpCSCC development.^(^ ^7^ ^,^ ^8^ ^)^

In most cases, oral SpCSCC presents as exophytic polypoid masses with an ulcerated surface,^(^ ^8^ ^)^ as observed in the present case. Sessile nodules and infiltrative ulcers are also observed with relative frequency in oral SpCSCCs.^(^ ^3^ ^,^ ^7^ ^)^ Lesions usually exhibit fast growth and may be associated with pain, dysphagia, odynophagia and bleeding,^(^ ^7^ ^)^ as in the case presented.

Microscopically, SpCSCC is characterized by a biphasic growth pattern, with proliferation of both squamous and spindle cells.^(^ ^9^ ^)^ The carcinomatous component may present as carcinoma *in situ*, similarly to the present case, or an invasive SCC among spindle cells.^(^ ^9^ ^)^ The sarcomatous component, which generally constitutes the major part of the lesion, may assume various patterns. In most cases, this component resembles fibrosarcomas and malignant fibrous histiocytomas.^(^ ^9^ ^)^ Microscopic distinction between SpCSCC and malignant mesenchymal tumors is difficult, or even impossible, without immunohistochemistry. In our case, it was essential to perform a large immunohistochemical panel to establish the diagnosis of SpCSCC.

The most useful epithelial markers for the diagnosis of SpCSCC include AE1/AE3, CK1, CK18 and EMA. Squamous areas are virtually always positive for AE1/AE3, whereas the spindle cell component reveals positivity in 40% to 85% of cases.^(^ ^3^ ^)^ The negativity for AE1/AE3 in some cases of SpCSCC emphasizes the importance of using other markers of epithelial cells, such as EMA and P63,^(^ ^10^ ^)^ in conjunction with pan-cytokeratin.^(^ ^9^ ^,^ ^10^ ^)^ In consonance with these suggestions, in our case, the sarcomatous component revealed negativity for AE1/AE3 and focal positivity for EMA and P63.

Spindle cells show positivity to vimentin and variable expression of other mesenchymal filaments, such as α-SMA, muscle-specific actin and desmin, whereas the carcinomatous component is negative for these markers.^(^ ^3^ ^,^ ^9^ ^)^ A similar profile of immunoreactivity was found in the present case. Positivity of the sarcomatous component to S-100 protein is observed only in a small number of cases of head and neck SpCSCC.^(^ ^3^ ^)^ Regarding Ki-67 expression, there was a positivity index in the spindle cells of approximately 40% in our case. A similar percentage (35%) was observed in the five oral SpCSCCs reported by Romañach et al.^(^ ^3^ ^)^

The treatment for head and neck SpCSCCs is total resection with a wide surgical margin.^(^ ^7^ ^,^ ^8^ ^)^ Despite the improvement in the local control of the disease with adjuvant radiotherapy,^(^ ^6^ ^)^ studies with univariate and multivariate analyses reveal absence of a significant impact of this therapeutic modality on the survival of patients with head and neck SpCSCCs when compared to isolated surgery.^(^ ^2^ ^,^ ^6^ ^)^

## CONCLUSION

Oral spindle cell squamous cell carcinoma is a rare, aggressive variant of squamous cell carcinoma that usually presents as an exophytic mass located on the tongue of elderly male patients. Microscopically, this malignancy may mimic other sarcomas and spindle cell malignancies; thus, immunohistochemistry is a valuable tool to establish the diagnosis of spindle cell squamous cell carcinoma.
